# Life‐Threatening Ectopic Pregnancy Implanted on the Uterosacral Ligament in a LNG‐IUS User: A Maternal Near‐Miss Event

**DOI:** 10.1155/crog/1602324

**Published:** 2026-03-06

**Authors:** Annalisa Di Cello, Jessica Sovereto, Maria Patrizia Muzzi, Carmelina Donatella Ermio

**Affiliations:** ^1^ Unit of Obstetrics and Gynecology, Department of Maternal and Child Health, Lamezia Terme Hospital, Azienda Sanitaria Provinciale (ASP), Catanzaro, Italy; ^2^ Unit of Obstetrics and Gynecology, Department of Clinical and Experimental Medicine, Magna Graecia University, Catanzaro, Italy, unicz.it

**Keywords:** ectopic pregnancy, emergency laparoscopy, hemoperitoneum, LNG-IUS, maternal morbidity, maternal near-miss, uterosacral ligament

## Abstract

**Background:**

Ectopic pregnancy is a major cause of first‐trimester maternal morbidity and mortality. Implantation on the uterosacral ligament is exceptionally rare and may lead to catastrophic intraperitoneal bleeding.

**Case Presentation:**

A 38‐year‐old woman using a levonorgestrel‐releasing intrauterine system (LNG‐IUS) presented in hemodynamic preshock with severe anemia and positive *β*‐hCG. Imaging revealed a large‐volume hemoperitoneum without identifiable intrauterine or adnexal gestation. Emergency diagnostic laparoscopy demonstrated approximately 5 L of hemoperitoneum and active bleeding from the right uterosacral ligament. The bleeding lesion was excised, and histopathology confirmed ectopic gestation. The patient recovered uneventfully and was discharged on postoperative Day 6.

**Conclusion:**

Uterosacral ligament ectopic pregnancy is an extremely rare but potentially fatal condition. This case constitutes a maternal near‐miss and underscores the importance of maintaining high suspicion and rapidly proceeding with surgical exploration when hemoperitoneum is present and diagnosis is unclear.

## 1. Introduction

Ectopic pregnancy remains one of the most serious complications of early gestation and an important contributor to first‐trimester maternal mortality worldwide [1, 2]. Although most ectopic pregnancies occur within the fallopian tubes, rare extratubal implantation sites have been described, including the cervix, cesarean scar, ovary, peritoneum, and uterosacral ligament (USL) [3, 4]. USL implantation is extremely uncommon, with estimates ranging from 1 in 10,000 to 1 in 30,000 pregnancies [5, 6].

Diagnosis is often challenging due to nonspecific symptoms and limited visualization on transvaginal ultrasound [7].

Although the levonorgestrel‐releasing intrauterine system (LNG‐IUS) is highly effective at preventing pregnancy, conception occurring with an IUS in situ is more likely to be ectopic compared with the general population [8, 9].

We present a rare and life‐threatening case of ectopic pregnancy implanted on the USL in a hemodynamically unstable LNG‐IUS user, fulfilling the criteria for a maternal near‐miss event.

## 2. Case Presentation

A 38‐year‐old Gravida 3 Para 2 woman presented to the emergency department with a sudden pelvic pain and 7‐day delayed menstruation. The patient denied recent sexual intercourse prior to symptom onset. She had been using a Kyleena LNG‐IUS for 2 years, with regular follow‐up confirming correct positioning.

On admission, she appeared pale, hypotensive (80/50 mmHg), and tachycardic (110 beats/min). Laboratory tests showed severe anemia (Hb 5 g/dL; Hct 20%) and *β*‐hCG of 390 mIU/mL. Resuscitation with packed red blood cells and plasma was initiated. Overall, the patient received six units of packed red blood cells and two units of fresh frozen plasma.

Transvaginal ultrasound demonstrated free intraperitoneal fluid, an LNG‐IUS in situ, and no intrauterine or tubal pregnancy. Transvaginal ultrasound was performed at admission; however, due to the massive hemoperitoneum and the absence of a clearly identifiable adnexal or intrauterine gestational sac, no suspicious ectopic lesion could be visualized.

CT confirmed massive hemoperitoneum.

Because of hemodynamic instability, emergency laparoscopy was performed 40 min after arrival. Approximately 5 L of blood and clots were evacuated (Figure 1). The uterus and adnexa were intact. Active bleeding originated from a fissured lesion medial to the right USL, extending toward the pararectal space (Figure 2). After senior gynecologic consultation, the lesion was excised; histopathology confirmed chorionic villi consistent with ectopic pregnancy.

Figure 1Massive hemoperitoneum into the abdominal cavity. Massive hemoperitoneum was observed at the beginning of the laparoscopic procedure. (a and b) Intraoperative view of clotted and free blood occupying the upper and lower abdomen.

**Figure:**
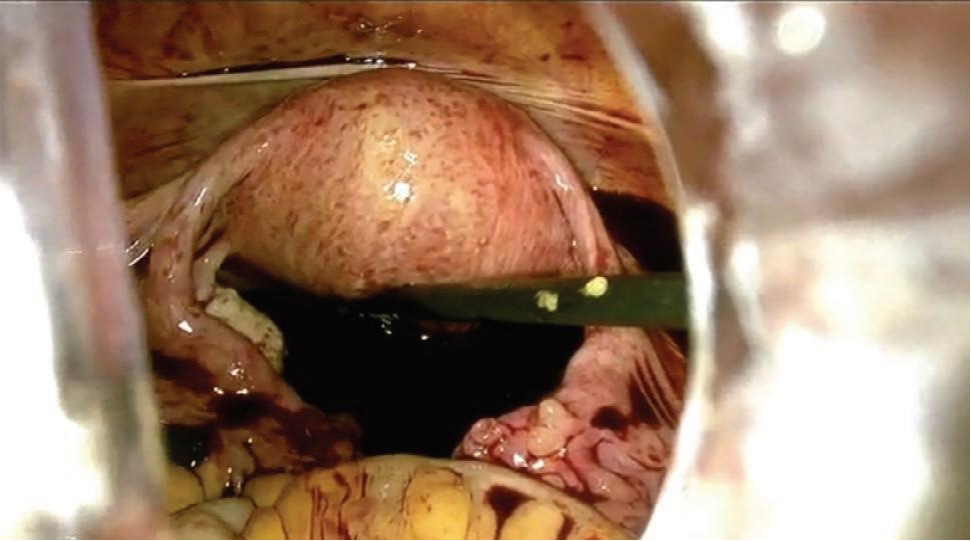
(a)

**Figure:**
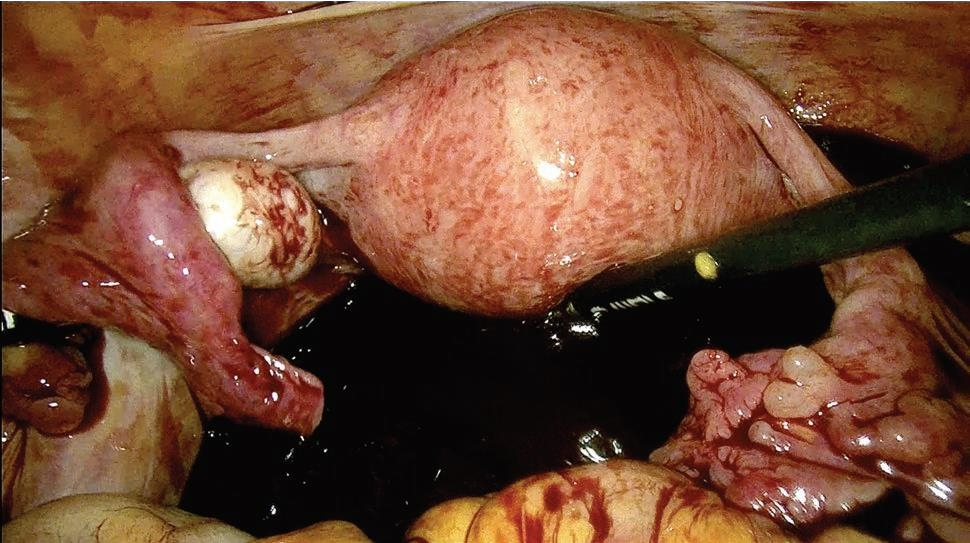
(b)

Figure 2The site of ectopic pregnancy implantation. Bleeding and fissured ectopic implantation site medial to the right uterosacral ligament (a and b). Laparoscopic identification of the ectopic pregnancy location after clot evacuation.

**Figure:**
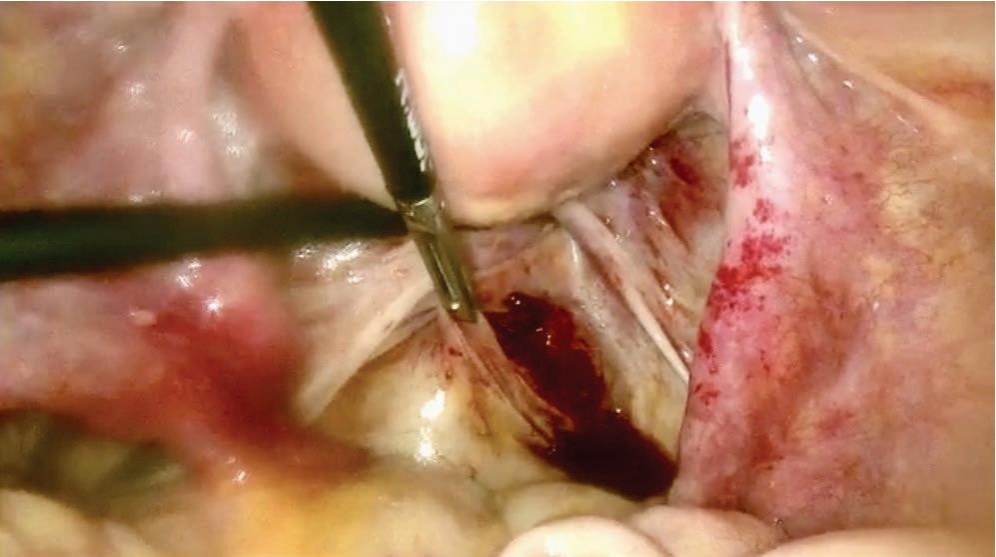
(a)

**Figure:**
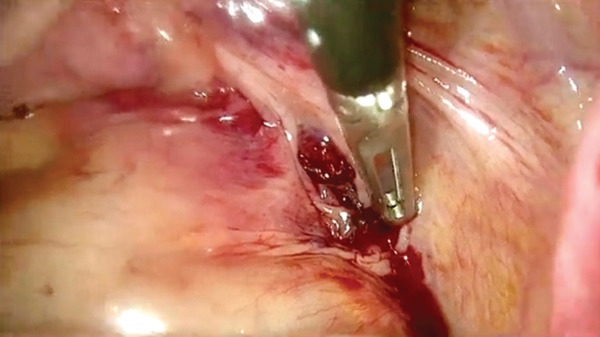
(b)

Hemostasis was obtained with bipolar coagulation and interrupted sutures. Rectal integrity was confirmed by hydropneumatic testing. A pelvic drain was placed. “The LNG‐IUS was removed after surgery once the patient was hemodynamically stable.”

Postoperatively, the patient was admitted to the ICU and received additional transfusions. *β*‐hCG levels peaked at 1600 mIU/mL before spontaneously declining. She was discharged on postoperative Day 6 without complications.

## 3. Discussion

USL implantation is one of the rarest forms of ectopic pregnancy and may lead to catastrophic bleeding due to proximity to the uterine and pelvic vessels [10, 11]. Diagnosis is often delayed because transvaginal ultrasound may fail to identify the lesion, as observed in this case

Abdominal ectopic pregnancy implantation within the pouch of Douglas has been hypothesized to result from early tubal abortion with secondary peritoneal implantation, facilitated by gravity, pelvic anatomy, and altered peritoneal receptivity. In rare cases, distinguishing true abdominal pregnancy from subserosal uterine implantation may be challenging, particularly when implantation occurs near the USLs. Recent literature has emphasized the importance of careful intraoperative assessment and histopathological confirmation to differentiate these entities, as subserosal pregnancies may mimic abdominal implantation but retain myometrial involvement [12]. In the present case, the absence of myometrial tissue at the implantation site and histological confirmation of chorionic villi support the diagnosis of true abdominal ectopic pregnancy.

Although LNG‐IUS markedly reduces overall pregnancy risk, any pregnancy occurring with an IUS in situ carries an increased relative risk of ectopic implantation [12–14]. More than half of such pregnancies have been reported to be ectopic in large cohort studies [15]. Lower‐dose LNG‐IUS devices (e.g., 13.5 mg) have slightly higher Pearl indices and ectopic pregnancy rates compared with higher dose systems [16].

Reported cases of USL ectopic pregnancy describe delayed diagnosis, occult bleeding, and acute presentations with hemoperitoneum [17–19]. Our patient met the WHO criteria for maternal near‐miss—survival from life‐threatening complications requiring urgent intervention [20].

This case demonstrates that emergency laparoscopy remains a life‐saving approach in the presence of hemoperitoneum and unclear diagnosis, consistent with ACOG guidelines for the management of ectopic pregnancy [21].

## 4. Conclusion

USL ectopic pregnancy is extremely rare but can rapidly become life‐threatening. Clinicians should maintain a high index of suspicion in hemodynamically unstable patients with positive *β*‐hCG and no identifiable intrauterine or adnexal pregnancy. Prompt surgical exploration is essential for reducing maternal morbidity and preventing mortality.

## Author Contributions

All authors contributed to patient management, data collection, and manuscript drafting.

## Funding

No funding was received for this manuscript.

## Disclosure

All authors gave final approval of the submitted version.

## Consent

Written informed consent was obtained from the patient for publication of this case report, including clinical details and images.

## Conflicts of Interest

The authors declare no conflicts of interest.

## Data Availability

The data that support the findings of this study are available from the corresponding author upon reasonable request.
